# Retrospective analysis of core-needle and vacuum-assisted breast biopsies of B3 fibroepithelial lesions and correlation with results in subsequent surgical specimens

**DOI:** 10.1007/s00432-024-05934-9

**Published:** 2024-09-28

**Authors:** Sophia Näther, Constanze Elfgen, Ann-Katrin Rodewald, Hisham Fansa, Heike Frauchiger-Heuer, Zsuzsanna Varga

**Affiliations:** 1 Department of Pathology and Molecular Pathology, University Hospital Zurich, Schmelzbergstrasse 12, CH-8091 Zurich, Switzerland; 2 Department of Internal Medicine, Spital Zollikerberg, Zollikerberg, Switzerland; 3 Breast Center Seefeld, Zurich, Switzerland; 4 University of Witten/Herdecke, Witten, Germany; 5 Breast Center, Spital Zollikerberg, Zollikerberg, Switzerland; 6 Breast Center, University Hospital Zurich, Zurich, Switzerland; 7 Comprehensive Cancer Center, University Hospital Zurich, Zurich, Switzerland

**Keywords:** Fibroepithelial tumor, Breast, Biopsy, Surgical specimen

## Abstract

**Background:**

Fibroepithelial lesions (FEL) are a heterogeneous group of biphasic tumours that include fibroadenomas (FA) and the rare entity of benign phyllodes tumors (PT) as well as cases where distinction between these two entities is not possible. The histologic distinction between benign PT and cellular FA is still a diagnostic challenge, especially in core-needle biopsy (CNB) or vacuum-assisted biopsy (VAB). Guidelines are not clearly established regarding the management of FEL in CNB or VAB. In this study, we addressed the frequency of B3 FEL diagnosed in CNB or VAB and compared the final histopathological findings in the excision specimens to evaluate up- or downgrading.

**Methods:**

We identified 117 female patients with the preoperative diagnosis of FEL (B3), PT, or FEL in combination of pure epithelial B3 lesions in CNB or VAB. Clinico-pathological information as well as data on subsequent surgical excision were available for all patients.

**Results:**

PT was diagnosed in 9 (14.8%) and FEL (B3) in 52 (85.2%) cases. Additionally, 56 patients with FA in combination with an additional B3 lesion were identified. Most FEL (B3)/PT initial diagnoses were made in CNB (55.6% of PT; 84.6% of FEL). After the initial biopsy, 7 of 9 (77.8%) patients with initial diagnosis of benign or borderline PT in CNB/VAB and 40 of 52 (77.0%) patients with initial diagnosis of FEL (B3) in CNB/VAB underwent open excision (OE). 4 of 9 cases (44.4%) initially diagnosed as PT were verified, whereas 2 of 9 (22.2%) were downgraded to FA. 20 of 52 cases (38.5%) initially diagnosed as FEL (B3) were downgraded to FA, whereas 11 of 52 cases (21.2%) were diagnosed as benign or borderline PT. One FEL (B3) case was upgraded to malignant PT.

**Conclusion:**

Most PT and FEL (B3) diagnoses on CNB/VAB underwent surgical removal. In the final pathological findings of cases classified primarily as FEL (B3), the majority were downgraded to FA, one quarter were upgraded to PT, and a small subset remained as combined FA/PT. In clinical daily practice, we recommend individualized decision-making considering different options (clinical follow-up or removal of the lesion depending on the whole context) in a multidisciplinary preoperative conference.

## Introduction

Fibroepithelial lesions (FEL) represent a heterogeneous group of biphasic tumors with stromal and epithelial components, including fibroadenomas (FA) as well as phyllodes tumors (PT) (Krings et al. 2017; Than et al. 2012). Pathomorphologically, findings are generally classified into five groups according to the B classification. B2 lesions, such as FAs, are indicative of benign entities, while B3 lesions, such as benign and borderline PT, signify benign lesions with uncertain biological potential (Leitlinienprogramm Onkologie and (Deutsche Krebsgesellschaft, Deutsche Krebshilfe, AWMF) 2021). While FA are relatively common B2 lesions, PT occur in only 0.3–1% of all breast tumors and in only 2.5% of all fibroepithelial tumors (Tse et al. 2019a). In contrast to FA, which predominantly affect women aged  < 30 years, PT primarily occur in middle-aged women, with an average age ranging between 40 and 50 years (Tse et al. 2019a; Co et al. 2018). Histologically, PT are characterized “by a double-layered epithelial component arranged in clefts surrounded by an often hypercellular stromal/mesenchymal component which in combination result in leaf-like structures” (Than et al. 2012). Based on various histological features, including the degree of stromal cellularity, mitoses, cytological atypia, stromal overgrowth, and the nature of tumor margins, PT are categorized as benign, borderline, or malignant (Than et al. 2012; Tse et al. 2019b; Varga et al. 2023).

The differentiation between cellular FA and benign PT is a diagnostic challenge, especially in CNB (Co et al. 2018; Ouyang et al. 2016; Shaaban and Barthelmes 2017; Youk et al. 2015; Zhou et al. 2016a, 2016b). The diagnostic difficulty arises from overlapping histological features of both entities. PT can be distinguished from FA primarily by their hypercellular stroma and the presence of leaf-like structures in the stroma (Tse et al. 2019a). Due to the high intratumoral heterogeneity of PT, some regions may look like FA, and the degree of cellularity may not be high enough for a PT diagnosis (Youk et al. 2015). The difficulty of distinguishing PT from FA has been confirmed in numerous interobserver studies, often revealing insufficient agreement in the diagnoses among different experts (Tse et al. 2019a; Dessauvagie et al. 2018; Lawton et al. 2014). In cases where FEL are histologically not distinguishable as FA or benign PT, a diagnosis of “benign epithelial neoplasm” can be made (Than et al. 2012; Elfgen et al. 2023).

Nonetheless, distinguishing between PT and FA, if possible, is clinically important because FA, depending on the size of the lesion, are managed conservatively or are therapeutically removed by VAE, while PT are mostly managed with open excision (OE) (Tse et al. 2019a; Dessauvagie et al. 2018). FA can also be surgically removed, especially large FA. As many patients complain about pain caused by the tumor they opt for removal. PT and FA have a low potential for local recurrence, with the overall rate of local recurrence for PT reported at 21% (Tse et al. 2019a). Considered separately, the recurrence rates range from 10 to 17% for benign PT, 14–25% for borderline PT, and 23–30% for malignant PT, while FA generally do not recur (Tse et al. 2019a). As of now, there are no definitive guidelines for the management of FEL that do not meet definitive criteria of PT or FA (Elfgen et al. 2023).

In this study, we investigated the frequency of FEL (B3) diagnoses in preoperative CNB and VAB samples, as well as the subsequent management decisions. Additionally, we were interested in the prevalence of concomitant epithelial B3 lesions which can occur within a FEL (especially in FA cases), resulting in a B3 category.

## Methods

The study is a retrospective, single-center clinical observational study in a cross-sectional design. Ethical approval was obtained from the local Ethics Committee of the Canton Zurich (ZH-KEK-2012-554). The study cohort includes female patients diagnosed with FEL (B3), PT, or an epithelial B3 lesion combined with FEL, through CNB or VAB of the breast between January 1, 2009 and December 31, 2013 using the clinical software “PathoPro” of the Institute of Pathology and Molecular Pathology of the University Hospital Zurich. There are two elements to this study. The first focuses on the diagnostic correlation between first and second line procedures in a series of pure FEL (B3) and PT. The second study component profiles the nature of accompanying B3 lesions in FEL. The pathological reports were queried for specific terms, including “B3 & phylloid*,” “B3 & fibroepithelial*,” “B3 & fibroadenom*,” “mamma & phylloid*,” and “mamma & fibroepithelial*.” The samples were collected from the participating Institutions (Breast Center of the University Hospital Zurich (USZ), the Breast Center of Spital Zollikerberg, and the Breast Center Seefeld). Data retrieval occurred through the utilization of the clinical software “PathoPro” and was subsequently anonymized before being consolidated in Microsoft Excel. Parameters captured in the dataset included gender, age at the time of the initial biopsy, biopsy method (CNB or VAB), histopathological findings from the biopsy, the subsequent procedure type (VAE/OE/clinical follow-up), and the final histopathological findings following VAE or OE.

## Results

### Cohort and initial diagnoses

**Fig. 1:**
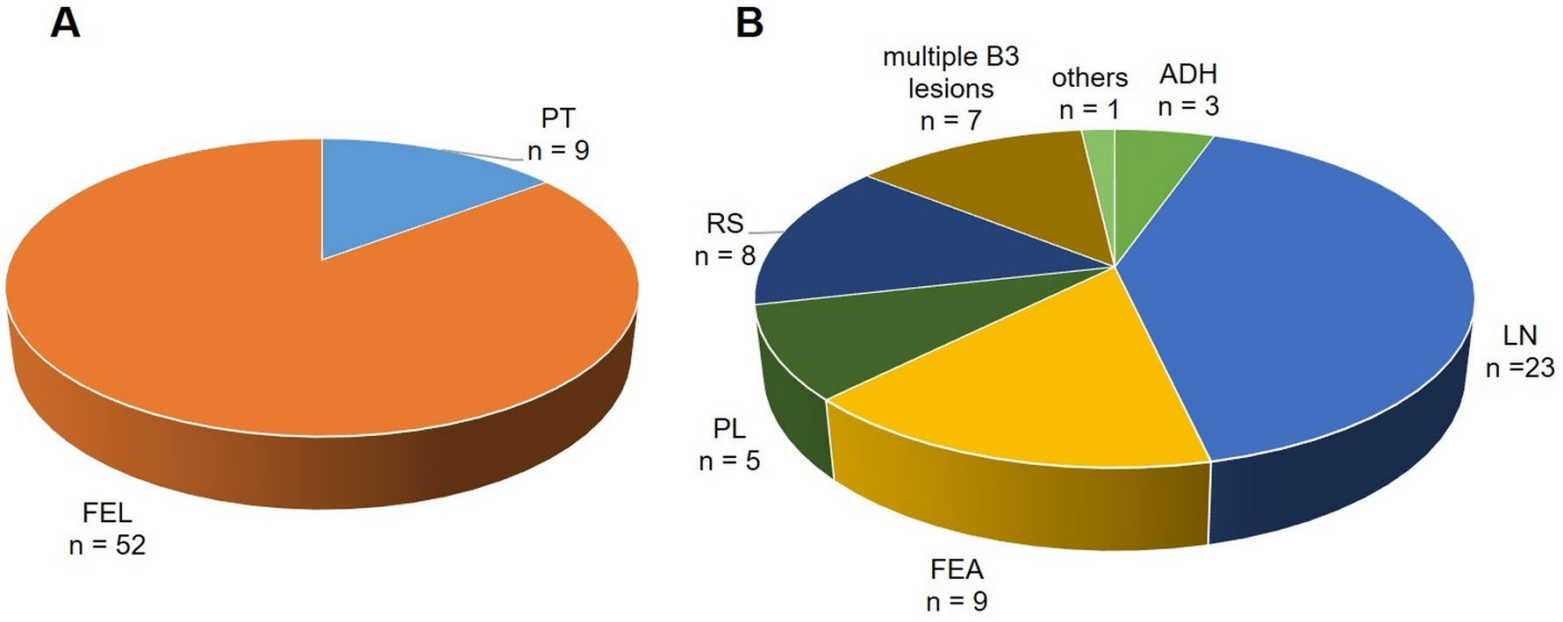
**A** Frequency of B3 FEL (without specification) and PT (B3), **B** Distribution of cases with FA in combination with additional B3 lesion(s). *FEL* fibroepithelial lesion, *PT* phyllodes tumor, *ADH* atypic ductal hyperplasia, *FA* fibroadenoma, *FEA* flat epithelial atypia, *LN* classical lobular neoplasia, *PL* papillary lesion, *RS* radial scar

The study cohort included 117 female patients aged between 16 to 78 (mean age: 44.5; standard deviation, SD: 16.4). Fibroepithelial B3 lesions (PT or FEL) were diagnosed in 61 patients. Among these cases, PT (benign or borderline) was initially diagnosed in 9 cases (14.8%). Histopathological findings in the remaining 52 women (85.2%) were reported as FEL (B3) without further specification (refer to raw data in Table 1 and graphical representation in Fig. 1A).

**Table 1:** Frequency of B3 FEL (without specification) and PT (B3)

	n *(%)*
PT	9 (14.8)
FEL	52 (85.2)
Overall	61 (100)

*FEL* fibroepithelial lesion, *PT* phyllodes tumor

The keyword search in PathoPro, as previously described, also identified 56 patients whose main histopathological report indicated a FA in combination with an additional B3 lesion. In most of these cases, patients had with classical lobular neoplasia (LN) combined with FA (n = 23; 41.4%). The second most frequent diagnosis was flat epithelial atypia (FEA) in combination with FA (n = 9; 16.1%). Radial scar (RS) combined with FA was observed in 8 cases (14.3%). Additionally, papillary lesions (PL) were present in 5 cases (8.9%), atypical ductal hyperplasia (ADH) in 3 cases (5.4%), and multiple B3 lesions combined with FA in 7 cases (12.5%). In one case classified as “others,” a clear diagnosis could not be established even after immunohistochemical verification. In this case, a fibroadenomatous change, some of which merges into cell-rich stroma, was described leaving the differential diagnosis of a cellular FS versus a desmoid-like fibromatosis, a definitive diagnosis could not be made using this material on VAB.

The distribution of cases involving FA in combination with an additional B3 lesion is presented in Table 2 and graphically illustrated in Fig. 1B*.*

**Table 2:** Distribution of cases with FA in combination with additional B3 lesion(s)

	n *(%)*
ADH (+ FA)	3 (5.4)
LN (+ FA)	23 (41.1)
FEA (+ FA)	9 (16.1)
PL (+ FA)	5 (8.9)
RS (+ FA)	8 (14.3)
Multiple B3 lesions (+ FA)	7 (12.5)
LN + RS	1
LN + PL	1
LN + FEA	3
ADH + FEA	1
ADH + PL + RS	1
Others	1 (1.8)
Overall	56 (100)

*ADH* atypic ductal hyperplasia, *FA* fibroadenoma, *FEA* flat epithelial atypia, *LN* classical lobular neoplasia, *PL* papillary lesion, *RS* radial scar

### Initial diagnostic procedures

#### CNB or VAB were performed as initial diagnostic procedures

**Fig. 2:**
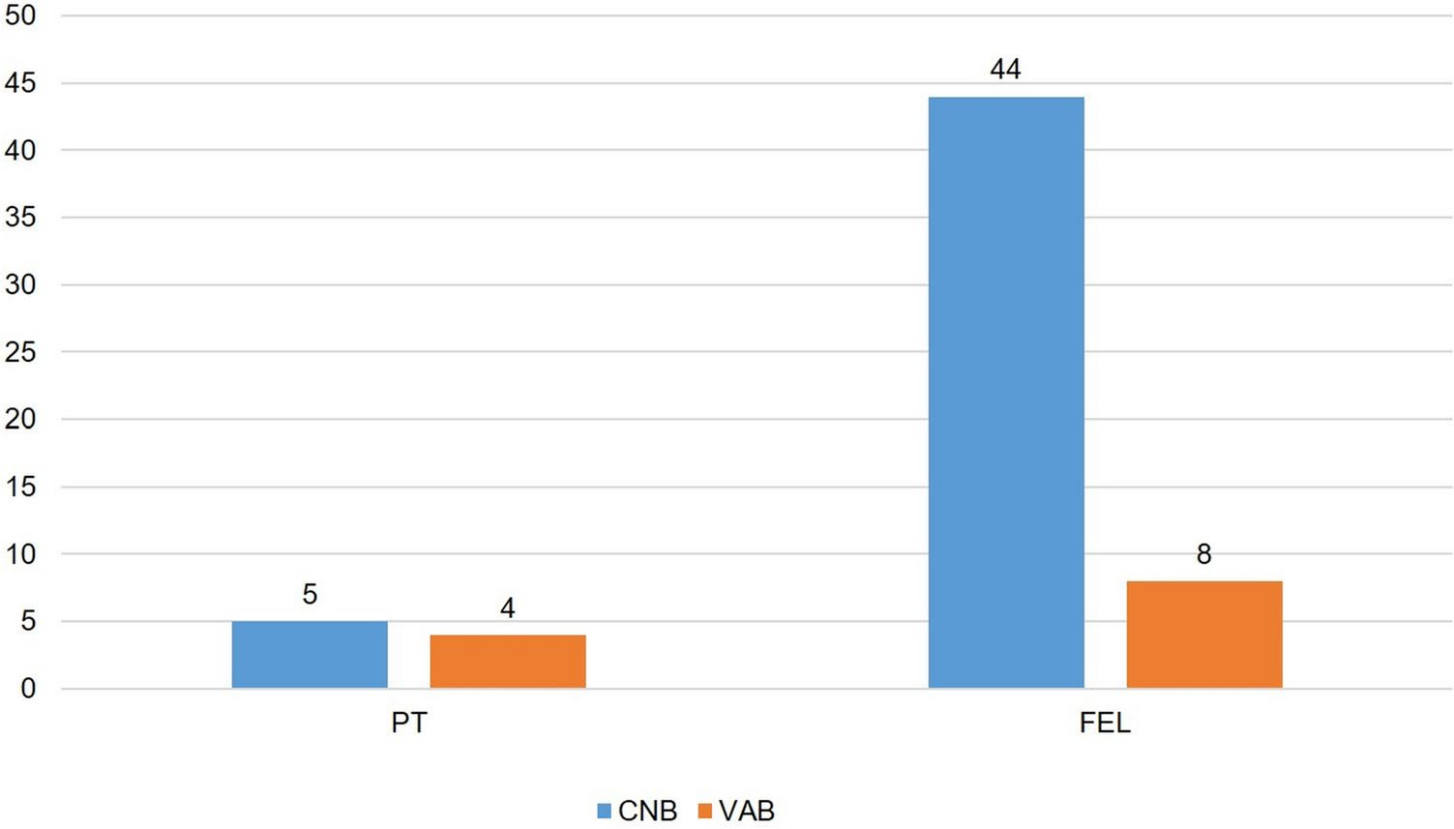
Diagnostic tools (CNB vs VAB) of B3 FEL (without specification) and PT (B3). *CNB* core-needle biopsy, *FEL* fibroepithelial lesion, *PT* phyllodes tumor, *VAB* vacuum-assisted biopsy

Within the subgroup of all fibroepithelial lesions (B3) (n = 61), the majority (n = 49; 80.3%;) of these lesions were diagnosed through CNB. Specifically, 5 PT (55.6%) were diagnosed through CNB, while the remaining PT cases (n = 4; 44.4%) were detected through VAB. For FEL (B3), 44 cases (84.6%) were diagnosed through CNB, and 8 cases (15.4%) through VAB. The details of initial diagnostic procedures for FEL are shown in Table 3 and Fig. 2*.*

**Table 3:** Diagnostic tool (CNB vs VAB) of B3 FEL (without specification) and PT (B3)

	Biopsy		Overall
	CNB	VAB	n *(%)*
	n *(%)*	n *(%)*	
PT	5 (55.6)	4 (44.4)	9 (100)
FEL	44 (84.6)	8 (15.4)	52 (100)
Overall	49 (80.3)	12 (19.7)	61 (100)

*CNB* core-needle biopsy, *FEL* fibroepithelial lesion, *PT* phyllodes tumor, *VAB* vaccum-assisted biopsy

**Fig. 3:**
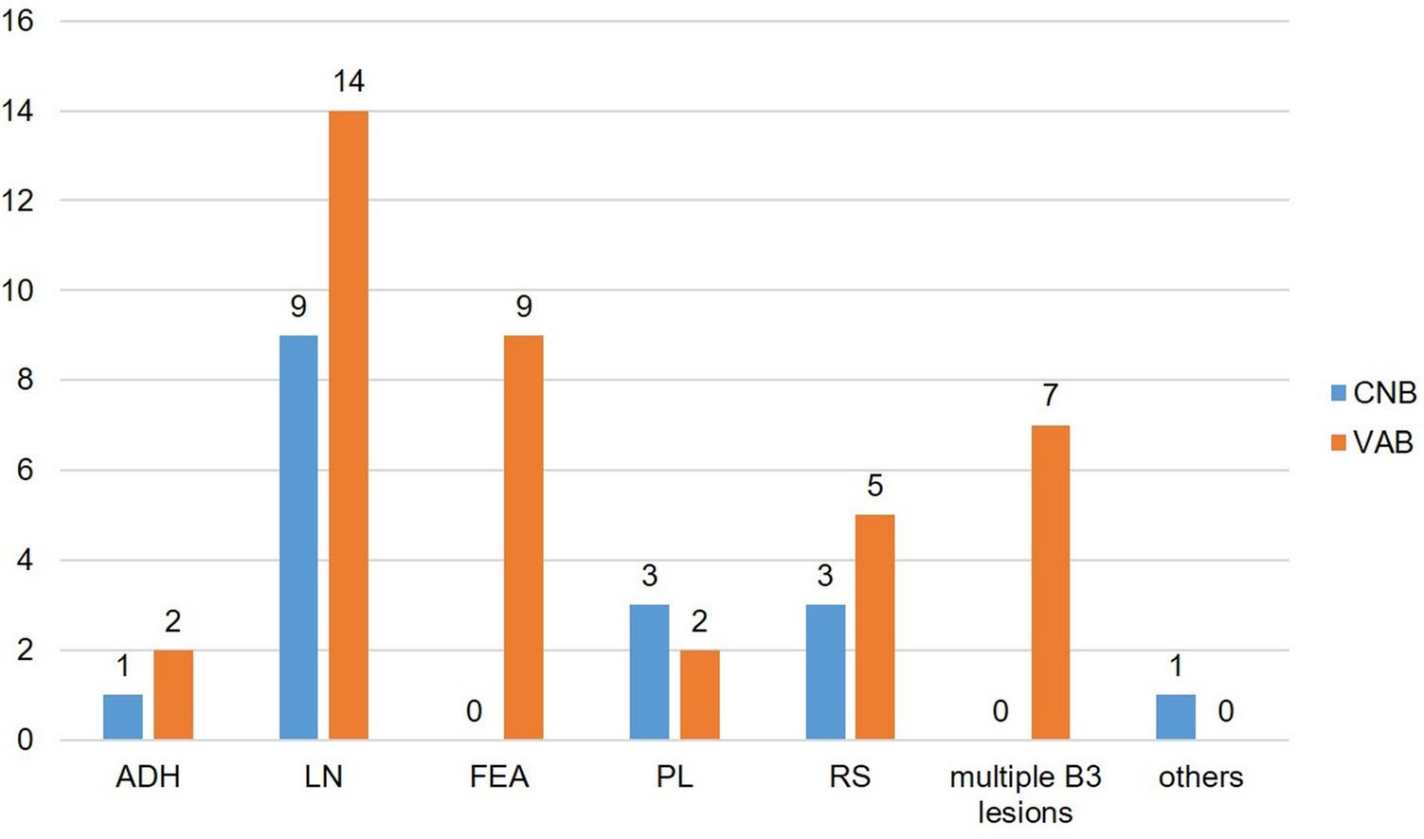
Diagnostic tool (CNB vs VAB) of FA with additional epithelial B3 lesion(s). *ADH* atypical ductal hyperplasia, *CAB* core-needle biopsy, *FA* fibroadenoma, *FEA* flat epithelial atypia, *LN* classical lobular neoplasia, *PL* papillary lesion, *RS* radial scar, *VAB* vacuum-assisted biopsy

In contrast, the majority (n = 39; 69.6%) of additional B3 lesions found in combination with FA (n = 56) were detected through VAB (Table 4 and Fig. 3). The remaining 17 (30.4%) B3 lesions in combination with FA were diagnosed through CNB.

**Table 4:** Diagnostic tool (CNB vs VAB) of FA with additional epithelial B3 lesion(s)

	Biopsy		
	CNB	VAB	Overall
	n (%)	n (%)	n (%)
ADH	1 (33.3)	2 (66.7)	3 (100)
LN	9 (39.1)	14 (60.9)	23 (100)
FEA	0 (0.0)	9 (100)	9 (100)
PL	3 (60.0)	2 (40.0)	5 (100)
RS	3 (37.5)	5 (62.5)	8 (100)
Multiple B3 lesions	0 (0.0)	7 (100)	7 (100)
Others	1 (100)	0 (0.0)	1 (100)
Overall	17 (30.4)	39 (69.6)	56 (100)

*ADH* atypical ductal hyperplasia, *CNB* core-needle biopsy, *FA* fibroadenoma, *FEA* flat epithelial atypia, *LN* classical lobular neoplasia,* PL* papillary lesion,* RS* radial scar,* VAB* vacuum-assisted biopsy

### Type of next clinical procedure

#### PT

Among the 5 PT cases initially identified through CNB, 4 patients (80%) subsequently underwent OE as the next diagnostic intervention, while one case underwent VAE (20%). For the 4 cases initially diagnosed through VAB, 3 underwent OE, and 1 patient had clinical follow-up (Table 5).

**Table 5:** Diagnostic procedure of PT in CNB and VAB

	Next procedure		
	VAB	OE	Clinical follow up
	n *(%)*	n *(%)*	n *(%)*
Biopsy			
CNB	1 (20.0)	4 (80.0)	0 (0.0)
VAB	0 (0.0)	3 (75.0)	1 (25.0)
Overall	1 (11.1)	7 (77.8)	1 (11.1)

*CNB* core-needle biopsy, *OE* open excision, *PT* phyllodes tumor, *VAB* vacuum-assisted biopsy

#### FEL (B3)

Of the 44 FEL (B3) initially diagnosed through CNB, OE was performed in 34 cases (77.3%), VAE in 2 cases (4.5%), VAE and OE in 3 cases (6.8%), and clinical follow up in one case (2.3%). Four patients were lost to follow up (9.1%). Out of the 8 FEL (B3) cases initially diagnosed through VAB, 3 patients (37.5%) underwent OE, and 3 patients (37.5%) underwent clinical follow-up. Two patients were lost to follow up (25%) (Table 6).

**Table 6:** Diagnostic procedure of FEL (B3) in CNB and VAB

	Next procedure				
	VAB	VAB + OE	OE	Clinical follow up	Lost to follow up
	n (%)	n (%)	n (%)	n (%)	n (%)
Biopsy					
CNB	2 (4.5)	3 (6.8)	34 (77.3)	1 (2.3)	4 (9.1)
VAB	0 (0.0)	0 (0.0)	3 (37.5)	3 (37.5)	2 (25.0)
Overall	2 (3.8)	3 (5.8)	37 (71.2)	4 (7.7)	6 (11.5)

*CNB* core-needle biopsy, *FEL* fibroepithelial lesion, *OE* open excision, *VAB* vacuum-assisted biopsy

### Additional B3 lesions in combination with fibroadenoma

**Fig. 4:**
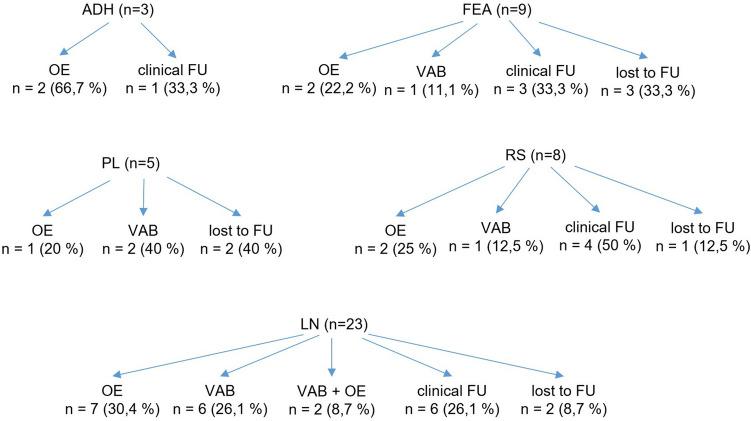
Type of next procedure for the individual B3 lesion(s) in combination with FA. *ADH* atypical ductal hyperplasia, *FEA* flat epithelial atypia, *FU* follow up, *LN* classical lobular neoplasia, *OE* open excision, *PL* papillary lesion, *RS* radial scar, *VAB* vacuum-assisted biopsy

OE, VAE, or clinical follow-up were individually decided and occurred with a similar frequency based on the type of epithelial B3 lesion and the clinical presentation. Figure 4 shows the type of next procedure for the individual B3 lesion in combination with FA.

### Final histopathological findings and downgrade or upgrade of initial diagnosis

#### Final pathological findings of cases classified primarily as benign or borderline PT

**Fig. 5:**
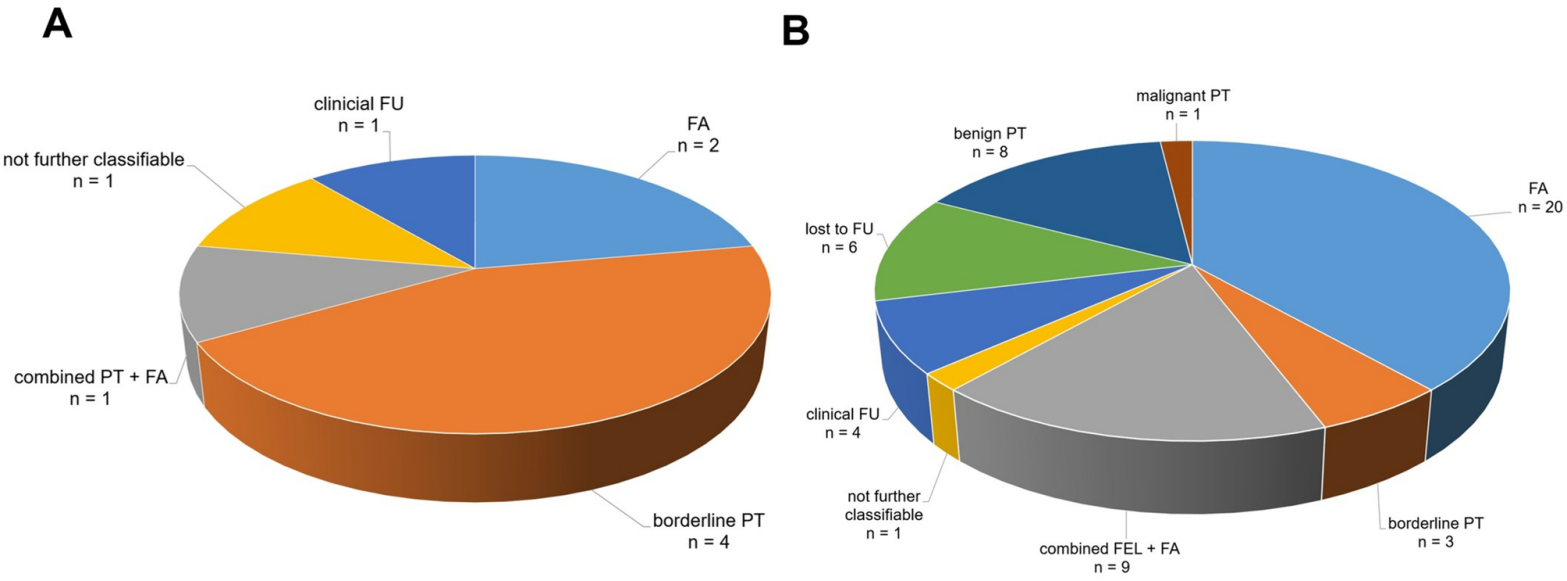
**A** Final pathological findings of cases classified primarily as benign or borderline PT (B3) in CNB and VAB, **B** Final pathological findings of cases classified primarily as FEL (B3). *FA* fibroadenoma, *FEL* fibroepithelial lesion, *FU* follow up, *PT* phyllodes tumor

Of the 9 patients diagnosed with PT, 8 underwent OE or a therapeutic VAE after initial CNB or VAB (88.9%). Among the 8 lesions excised, definitive histology revealed 2 FA (22.2%) and 4 borderline PT (44.4%). Thus, 2 cases (22.2%) were downgraded (mean age: 48.0), and 4 PT (44.4%, mean age: 38.8) were verified with OE or VAE. No benign PT were identified and no upgrade to malignancy was observed. One case involved a combined fibroepithelial lesion (PT + FA), and one case could not be further classified (Table 7*, *Fig. 5A).

**Table 7:** Final histopathological findings in surgical specimens (VAE and OE) of cases classified primarily as fibroepithelial lesions (PT + FEL, B3)

	PT	FEL
	n *(%)*	n *(%)*
FA	2 (22.2)	20 (38.5)
Benign PT	0 (0.0)	8 (15.4)
Borderline PT	4 (44.4)	3 (5.8)
Malignant PT	0 (0.0)	1 (1.9)
Combined FEL/PT + FA	1 (11.1)	9 (17.3)
Not further classifiable	1 (11.1)	1 (1.9)
Clinical follow up	1 (11.1)	4 (7.7)
Lost to follow up	0 (0.0)	6 (11.5)
Overall	9 (100)	52 (100)

*FA* fibroadenoma, *FEL* fibroepithelial lesion, *PT* phyllodes tumor, *VAE* vacuum assisted excision, *OE* open excision

#### Final pathological findings of cases classified primarily as FEL (B3)

Out of 52 patients diagnosed with FEL (B3), 42 underwent OE or therapeutic VAE after initial CNB or VAB (80.8%). Among the 42 lesions excised, pathology revealed 20 FA (38.5%) (downgrade, mean age: 31.6), 8 benign PT (15.4%, mean age: 36.1), and 3 borderline PT (5.8%, mean age: 60.7). Thus, twelve FEL (B3) (23.1%) were verified as PT with OE or a VAE. One malignant PT was identified, resulting in one upgrade case (age: 44). A combined fibroepithelial lesion (FEL + FA) was diagnosed in 9 cases (17.3%), and one case was not further classifiable (1.9%) (Table 7 and Fig. 5B).

**Fig. 6:**
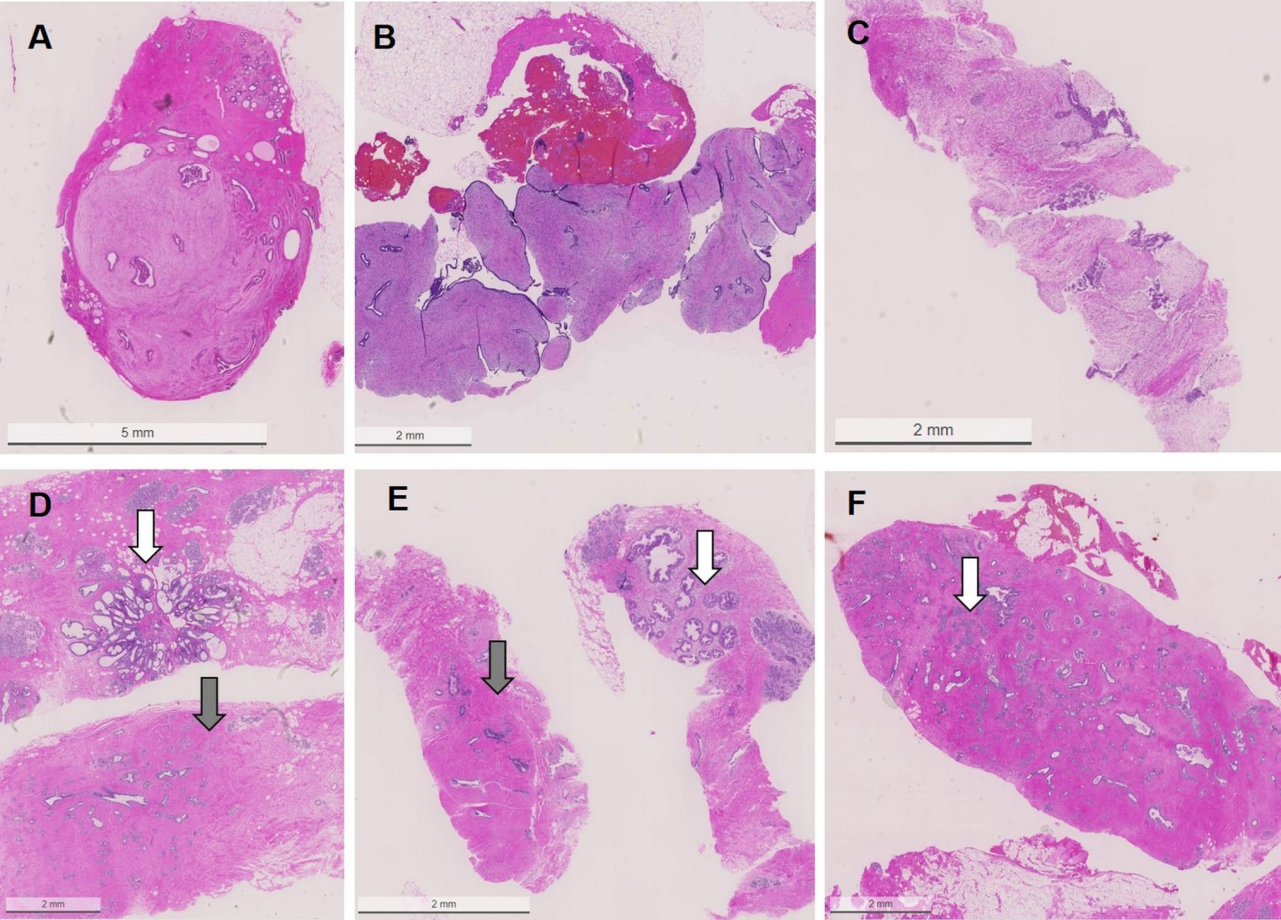
Histological presentations of fibroepithelial lesions in with or without further atypical lesions. **A** fibroadenoma in VAB, **B** phyllodes tumor in CNB, **C** fibroepithelial lesion without specification in CNB, **D** fibroadenoma (gray arrow) and a radial scar (white arrow) in VAB, **E** fibroadenoma (grey arrow) and a separate ADH (white arrow) in VAB, **F** fibroadenoma (grey arrow) with ALH (white arrow) in VAB

Histological presentation of the individual lesions are shown in Fig. 6.

## Discussion

In the present study, we examined the post-diagnostic management of B3 FEL identified through CNB or VAB and compared the final histopathological outcomes following excision with the initial biopsy diagnosis. Of particular interest was identifying cases of up- or downstaging post-surgery and characterizing histological diagnoses in excisions initially diagnosed as FEL (B3).

Histologically, a diagnosis of FEL (B3) was established in the biopsy (CNB or VAB) of 61 patients. In a majority of these cases, the histological classification could not definitively distinguish between FA or PT, prompting the assignment of a diagnosis of FEL (B3). In these cases where a definitive diagnosis could not be made, the recommendation to use the term “benign fibroepithelial lesion, B3” was applied (Than et al. 2012). The large proportion of those biopsies diagnosed as “FEL” underlines the challenge in histological distinction between FA and benign PT (Tse et al. 2019a; Co et al. 2018; Ouyang et al. 2016; Shaaban and Barthelmes 2017; Youk et al. 2015; Zhou et al. 2016a, 2016b).

Leaf-like growth is the best discriminator between FA and benign PT but is not always well-developed in PT. When a FEL shows other features of PT, a lack of leaf-like structures should not exclude the diagnosis. On the other hand, leaf-like areas may also be seen in FA, but these are typically focal and less cellular without stromal condensation (Krings et al. 2017).

Among PT and FEL (B3) cases initially diagnosed in CNB within our cohort, the majority (80% of PT, 77.3% of FEL, B3) underwent OE as the subsequent diagnostic or therapeutic step. Compared to other studies, this represents a relatively high proportion of OE. However, the numbers also vary in the literature. Marcil et al. reported a 64.9% rate of OE in patients initially diagnosed with FEL through CNB (Marcil et al. 2017), while other studies report lower rates (17%, 43%) (Resetkova et al. 2010; Osdol et al. 2014), opting for clinical and/or imaging follow-up in remaining cases.

Regarding the clinical procedure following the diagnosis of PT or FEL (B3), there are no uniform guidelines. The current National Comprehensive Cancer Network (NCCN) guideline recommends VAB as the next diagnostic step for indeterminate FEL (B3) or benign PT in a CNB (NCCN 2023). If benign PT is found on VAB, clinical follow-up for 3 years is recommended (NCCN 2023). This contrasts with the recommendations of the Third International Consensus Conference (Elfgen et al. 2023). The majority of the 33 participating specialists recommended OE after CNB diagnosis of PT. If a B3 PT diagnosis is made on VAB, the option of OE or clinical follow up are both justified if the lesion is radiologically removed (45% OE vs. 55% no intervention) (Elfgen et al. 2023). Patients with benign PT who were treated by VAB alone seem not to have a more compromised relapse-free survival than those who underwent OE (Ouyang et al. 2016). Sars et al*.* examined the prevailing clinical approaches to PT management through an international cross-sectional study and identified significant variability in clinical practices. This can also result in an overtreatment of many patients (Sars et al. 2023).

Our final histological findings reveal that cases initially classified as benign or borderline PT on biopsy were downgraded to FA in 22.2% of cases. The remaining cases either remained borderline PT (44.4%) or were given the category unclassifiable FEL (B3). Exact comparisons of our findings with literature data are difficult due to different study designs. In general, CNB often fails to diagnose PT (Youk et al. 2015). The rate of concordance of PT grade between CNB and surgery is approximately 60%. All discordant cases were firstly underestimated in CNB (Choi and Koo 2012).

In cases initially classified as FEL (B3), nearly half of the excised lesions were reclassified as FA after OE (20 of 42). This finding that most FEL (B3) diagnosed by CNB turn out to be FA after OE aligns with findings from other studies (Marcil et al. 2017; Resetkova et al. 2010; Mohan et al. 2022). Marcil et al. und Resetkova et al. found FA in 62.5% respectively 53% of cases who underwent surgery after initial diagnosis of FEL (B3) in CNB (Marcil et al. 2017; Resetkova et al. 2010). However, Mohan et al. found in their 10-year single-center study an upgrade rate from FEL (B3) on CNB to PT upon excision in 25.8%, advocating for FEL (B3) excision (Mohan et al. 2022).

The distribution of PT in our series is similar to existing literature data, with benign PT being the most common diagnosis (15.4%), followed by borderline (5.8%) and malignant PT (1.9%) (Co et al. 2018; Youk et al. 2015; Tan et al. 2012).

In our series, we found a total of 56 cases in which additional B3 lesions occurred in combination with FA. Regarding further management of these lesions, there was no clear tendency towards a conservative or surgical approach. Literature data for comparison are not available for these special findings when an epithelial B3 lesion occurs together with a FA. In general, the decision regarding further diagnostic and therapeutic management is individual, should be discussed interdisciplinary, and is mainly driven by the given epithelial B3 lesion independently of the presence of FA.

In conclusion, our data highlight key considerations for the clinical management of FEL (B3) and PT. In the absence of standardized guidelines, individualized decision-making is crucial for FEL (B3) diagnosed in CNB or VAB. In general, studies as well as current recommendations show that a CNB with a diagnosis of FEL (B3) should at least be supplemented by a VAB, as the diagnosis can only be made to a limited extent in the CNB. Our study indicates a predominant utilization of OE as the subsequent diagnostic step. Notably, downgrading from FEL (B3) to FA was observed in many cases. FA arising with concomitant epithelial B3 lesions are managed mainly based on the individual accompanying B3 lesion. These data can be used to advise patients based on the results of the initial biopsy and to indicate possible prognoses. Above all, it is important that a multidisciplinary preoperative conference guides further diagnostic and therapeutic decisions (Elfgen et al. 2023).

From the pathologist’s perspective, our data show evidence in favor of a more conservative approach in the further management of FEL (B3).

While our study has limitations, including its retrospective design and a relatively small case number due to the rarity of PT and FEL (B3) (Than et al. 2012), it provides valuable insights into the prevailing clinical practices. Some cases were lost to follow-up. Larger, case–control studies with extended follow-up durations could aid the decision whether to undergo surgical excision or continue clinical surveillance of FEL (B3) found in CAB or VNB.

In summary, this single-center study shows that most benign or borderline PT or FEL (B3) found in CNB or VAB are surgically removed. In the final pathological findings of cases classified primarily as FEL (B3), most cases were downgraded to FA. Even in the surgical specimen, some findings cannot be clearly assigned to an entity.

## Data Availability

No datasets were generated or analysed during the current study.

## Abbreviations

ADH: Atypical ductal hyperplasia
CNB: Core-needle biopsy
FA: Fibroadenoma
FEA: Flat epithelial atypia
FEL: Fibroepithelial lesion (including benign epithelial tumor not clearly classifiable as fibroadenoma or phyllodes tumor)
LN: Classical lobular neoplasia
OE: Open excision
PL: Papillary lesion
PT: Phyllodes tumor
RS: Radial scar
VAB: Vacuum-assisted biopsy
VAE: Vacuum-assisted excision
